# Highly suspected cases of salmonellosis in two cats fed with a commercial raw meat-based diet: health risks to animals and zoonotic implications

**DOI:** 10.1186/s12917-017-1143-z

**Published:** 2017-07-24

**Authors:** Federica Giacometti, Jacopo Magarotto, Andrea Serraino, Silvia Piva

**Affiliations:** 1 Department of Veterinary Medical Sciences, University of Bologna, Via Tolara di Sopra 50, 40064 Bologna, Italy; 2 Veterinary practitioner, 30174 Venice, Italy

**Keywords:** *Salmonella* sp., Cat, Raw meat-based diet, Zoonotic risk

## Abstract

**Background:**

Feeding raw meat-based diets (RMBD) to companion animals raises public health concerns for both animals and humans. While considerable attention has been paid to bacterial contamination of commercial pet food, few literature studies have investigated foodborne disease in companion animals. Salmonellosis is reported to be infrequent in cats but no known data or studies estimating feline salmonellosis are available or large-scale epidemiological studies assessing *Salmonella* risk factors.

**Case presentation:**

Two highly suspected cases of salmonellosis in two cats fed with a commercial frozen poultry RMBD are presented, for the first time from the same household. The clinical presentation, diagnostics, treatment and follow-up are reported and the zoonotic implications are discussed.

**Conclusions:**

This case highlights the health risks posed to both animals and owners by feeding RMBD to pets, and suggests that these risks should be considered by veterinary practitioners.

## Background

Non-typhoidal *Salmonella* spp. are important human and animal pathogens worldwide. Most human salmonellosis cases are foodborne. However infections are also reported through direct or indirect animal contact in homes, veterinary clinics, zoological gardens, farms or other public, professional or private settings [1]. Incidents of human salmonellosis in Europe (EU) are relatively common (EU notification rate of 23.4 cases per 100,000) and most are attributed to the consumption of contaminated foods (55%) but fairly high percentages are linked to environmental sources (13%) and even to direct animal contact (9%) [2]. Likewise, pets are also susceptible to *Salmonella* infection, but no known data or studies estimating salmonellosis cases in pets are available.

Of the primary enteropathogenic bacteria, *Salmonella* spp. is found in the feline intestinal tract [3–5]; salmonellosis is uncommon in cats, often, favoured by host immunosuppression [6]. Cats are primarily infected subclinically, but gastrointestinal disease manifested as enterocolitis and endotoxemia can occur and is initially associated with fever, malaise, and anorexia followed by vomiting, abdominal pain, and diarrhoea [7]. Abortion, stillbirth, meningoencephalitis, respiratory distress and conjunctivitis have also been described [6, 8]; the disease manifestations range from mild self-limited diarrhoea to potential and fatal gastroenteritis and septicaemia [9].

*Salmonella* prevalence among cats is variable and similar in diarrhoeic and non-diarrhoeic cats, with shedding rates from 0 to 8.6% in diarrhoeic cats and from 0 to 14% in non-diarrhoeic cats [7, 10–12]. However, higher prevalence rates are found in stray or shelter dogs/cats and in dogs fed with raw food diets with the odds of shedding *Salmonella* estimated to be 23 times greater for dogs fed with raw food diets than those given commercial diets [13–16].

Feeding raw diets to domestic cats is becoming increasingly popular and today there are many options for owners who wish to change their feeding practices. Recipes for home-made raw food diets are widely available and commercial frozen food diets are also sold at some pet stores and veterinary clinics. The main ingredients of these products are raw meat, mainly poultry and beef, vegetables, grains and fruits [17]. As these diets do not undergo any type of heat processing or sterilization, existing bacteria and parasites can be present at the time of consumption [15] so both commercial and home-made raw meat-based diets (RMBD) are at risk of contamination with pathogens including *Salmonella* spp. [17, 18].

*Salmonella spp.* are transmitted directly or indirectly by the faecal-oral route [13]. Sources of infection with *Salmonella* spp. for indoor cats include the ingestion of raw meat and some processed food, while outdoor cats are at risk from scavenging and hunting rodents and birds, exposure to reptiles and environmental contamination [8, 9, 17, 19]. However, large-scale epidemiological studies assessing *Salmonella* spp. and their risk factors are lacking with only one report to date describing two cases of feline salmonellosis in a multicat household fed with a home-made contaminated beef RMBD [9].

This report describes two highly suspected cases of feline salmonellosis in two cats from the same household fed with RMBD and identifies a likely source of the *Salmonella* cases.

## Case presentation

### Case number 1

In January 2014, an 8-year-old neutered female indoor domestic cat, Sphynx breed, was evaluated at a private veterinary clinic for gastrointestinal signs, namely anorexia with recent weight loss, vomiting and diarrhoea. The signs had began 10 days earlier with lethargy/weakness and malaise; the cat had a history of gastrointestinal and hepatic problems. For most of the cat’s adult life, her body weight had been consistent at approximately 4 kg. The cat was housed with one other cat, her daughter, which reported no signs. At the first visit, the owner reported giving the animals the same diet, namely homemade and commercial dry pet food.

On physical examination, the cat weighed 2.8 kg, the rectal temperature was 39.5 °C, pulse rate was 180 beats/min, and respiratory rate was 36 breaths/min; abnormalities on physical examination included fluid-splashing sounds in the abdomen, about 8% dehydration, left retromandibular lymph node enlargement, and the oral examination showed gingivitis, plaque and halitosis. The cat appeared otherwise normal.

Faecal examination showed a mucoid and bloody diarrhoea and the faecal flotation test and SNAP faecal enzyme-linked immunosorbent assay (ELISA) *Giardia* test (SNAP *Giardia* test, IDEXX Laboratories, Maine, USA) were negative. A complete geriatric haemobiochemical profile was sent to Idexx Laboratory; results of the cell blood count (CBC), venous haemogas analysis, serum biochemical analysis and assessment of serum total thyroxine concentration were within reference intervals except for leukocytes (23.6 G/l, reference interval, 6–11 G/l), absolute segmented neutrophils (19,030/ul, reference intervals 3000-11,000/ul), absolute monocytes (1676/ul reference intervals 0-500/ul); ALT (256 U//l, reference interval, < 175 U/l), AST (118 U/l, reference intervals <71 U/l) serum albumin concentration (17 g/l, reference intervals 27–44 g/l), calcium (1.9 mmol/l, reference interval, 2.2-2.9 mmol/l), and folate (7.9 ng/ml, reference interval 11.1 - 21.6 ng/ml).

A diagnosis of generic enteritis, hepatopathy with probable intestinal infection, was made. The cat was hospitalized for 6 days and treated intravenously with: fluid therapy (Ringer’s acetate), Metronidazole antibiotic (10 mg/kg bid - Deflamon 500 mg/100 mL), Enrofloxacin antibiotic (5 mg/kg sid - Baytril 50 mg/ml), S-Adenosylmethionine (20 mg/kg sid - Samyr 400 mg/5 ml). The cat was fed with a gastrointestinal diet (Prescription Diet i/d Feline, Hill’s) and lactobacillus (Florentero, Candioli Pharma), and treated with milbemycin oxime and praziquantel (Milbemax®, Novartis).

### Case number 2

Four days after case number 1 was seen for the first time, a 6-year-old female cat from the same household was referred to the same veterinary clinic for the same signs previously reported for case number 1, 3 days of vomiting and diarrhoea and 1 day of anorexia, but milder than case 1. For most of the cat’s adult life, her body weight had been approximately 3 kg.

During the clinical visit and considering the anamnesis of case number 2, the veterinarian suspected the same infection. Investigating the infection and the possible sources of contamination more deeply, the owner specified that the homemade food given to both animals was a frozen commercial poultry RMBD bought on the internet.

On physical examination, the cat weighed 2.5 kg, the rectal temperature was 38.3 °C, pulse rate was 180 beats/min, and respiratory rate was 40 breaths/min; abnormalities included only about 7% dehydration. Faecal examination showed a mucoid diarrhoea and the faecal flotation test was negative.

Clinicopathologic findings (procyte Idexx, catalyst Idexx), CBC and restricted serum biochemical analysis were within reference intervals except for leukocytes (22.14 K/ul, reference interval, 2.87-17.02 K/ul), absolute segmented neutrophils (18.2 K/ul, reference intervals 1.48-10.29 K/ul), absolute monocytes (0.72 K/ul reference intervals 0.05-0.67 K/ul); ALT (132 U//l, reference interval, < 130 U/l), AST (57 U/l, reference intervals <48 U/l) and GGT (4 U/l, reference intervals 0–1 U/l).

Based on the information acquired during anamnesis of the second case and the fact that both cats had received the same raw food diet, fresh faeces of case number 2 were collected and sent to IDEXX Laboratories for a real-time PCR assay evaluating a panel of 8 enteropathogens (Feline Diarrhoea RealPCR™ Panel) including feline panleukopenia virus, feline coronavirus, *Tritrichomonas foetus*, *Giardia* sp., *Toxoplasma gondii*, *Cryptosporidium* sp., *Salmonella* sp. and the detection of *Clostridium perfringens* toxin A gene (*cpa*) and *Clostridium perfringens* enterotoxin gene (*cpe*). The PCR assays detected *Salmonella* sp. and 1,300,000 copies/g of *cpa* were quantified; no other enteropathogens were detected. The cat was hospitalized for 4 days and treated as reported for case number 1.

### Case follow-up

At the time of writing, a diagnosis of inflammatory bowel disease was made for case number 1 and the cat reports several daily episodes of diarrhoea, whereas case number 2 is healthy and never showed any recurrence of gastrointestinal signs.

### Analysis of commercial frozen poultry RMBD and commercial dry pet food

On the basis of the *Salmonella* sp. and *C. perfringens* positive faeces, the owner of the two cats was advised by the veterinarian to discontinue the practice of feeding raw meat-based diets and the commercial poultry RMBD was suspected as a possible source of infection. The owner submitted specimens of both diets, namely commercial prepared bowl of frozen poultry RMBD and commercial dry pet food, to the Experimental Institutes for Zooprophylaxis in Veneto for bacteriological culture: the diets were analyzed for the detection of *Salmonella* sp. with real-time PCR (iQ-Check® *Salmonella*, Bio-Rad) validated by AFNOR (BRD 07/06-07/04) and also using the official International Organization for Standardization (ISO) cultural methods, ISO 6579:2002/Cor 1:2004, and using the ISO 7937:2004 for the count of *C. perfringens*. The RMBD results disclosed the presence of *Salmonella* spp. DNA by real-time PCR and the isolation of *Salmonella* spp., that was serotyped as *Salmonella* Typhimurium Group B 1,4,(5),12:i:1,2, whereas *C. perfringens* was counted as <10 colony forming units (cfu)/g. *Salmonella* spp. was not detected by real-time PCR in the commercial dry pet food, and *C. perfringens* was counted as <10 cfu/g.

## Disc﻿ussion and conclusions

Diarrhoea is common in domestic cats, occurring as a result of gastrointestinal disease (including dietary causes, gastrointestinal infection, inflammation or neoplasia) or extra-intestinal disease, and the diagnosis can be frustrating for clinicians and owners alike [3]. The traditional diagnosis of feline salmonellosis is based on the isolation of *Salmonella* spp. in conjunction with clinical signs and assessment of the potential risk factors because the isolation of *Salmonella* from cats alone can be insufficient [7]. In this report, the culture results of RMBD, the clinical signs of the two cats, the detection of *Salmonella* spp. DNA in cat number 2 and the exclusion of other potential aetiological agents (with the exception of *C. perfringens* toxin A gene detection) suggest this is a case of salmonellosis.

In relation to the *C. perfringens* toxin A gene detected in the faeces of case number 2, *C. perfringens* biotype A is the most common genotype in dogs and cats and part of the normal canine intestinal microflora [7]. Its role as an enteropathogen is not fully understood and it is suspected to be associated with anything from mild, self-limiting diarrhoea to rapidly fatal necrohaemorrhagic enteritis. Most studies indicate this as a primary enteropathogen but some authors suggest it could act as an opportunistic agent in dogs and cats [20]. Disruption of the normal microbiota, such as a sudden change to a high protein diet, or enteric infection by other pathogens, like parvovirus, are considered predisposing factors in dogs [11, 21]. Diagnosis of *C. perfringens* type A associated diarrhoea in companion animals is challenging because the clinical signs cannot be differentiated from enteritis caused by other enteropathogens. Recent studies correlating the presence of *cpe* in feces with diarrhoea in dogs suggest that the detection of this toxin in stool samples could be useful to diagnose *C. perfringens* type A-associated diarrhoea. However, no study has confirmed the role of the *C. perfringens* enterotoxin in dogs and both enterotoxin and isolates positive for the *cpe* gene can be found in healthy dogs, so these methods could suggest that *C. perfringens* is involved, but they are not confirmatory [20]. Similar observations are reported to explain the results from the IDEEX available for case no. 2. The notes linked to *cpa* and *cpe* laboratory results highlight that the presence of the DNA of the *C. perfringens* alpha toxin over 300,000 copies/g could be implicated in the clinical signs but, in case of *cpe* copies under the limit or absent, as in our case, it is unlikely that *cpa* is the cause of diarrhoea and definitely not *cpe*. All these aspects led us to speculate that a co-infection was responsible*.* It is important to note that unlike studies in dogs, no association has been found between diarrhoea and detection of the enterotoxin in stool samples or detection of *cpe* in *C. perfringens* strains isolated from cats. Hence, the diagnosis should be based only on the isolation of *C. perfringens* (positive or negative for *cpe*) in conjunction with the absence of other enteropathogens [20].

The detection of *Salmonella* spp. DNA in case number 2 faeces and the isolation of *Salmonella* in the commercial RMBD support the hypothesis of a *Salmonella* infection caused by the consumption of contaminated RMBD. However, no cultural examinations were performed in faeces of the two cats to avoid isolates from animals and feed being characterized by a discriminative typing method, therefore representing a major limitation of our findings. Considering that the sources of *Salmonella* exposure are dependent on whether cats are indoor or outdoor pets [17] and that both cats were households pets with no contact with other animals or the outside environment, the most likely source of exposure is consumption of food contaminated with *Salmonella.* In this context, veterinary practitioners should be trained and encouraged on the need for faecal culture in case of positive PCR findings in order to optimize the identification and management of enteropathogenic bacteria in companion animals [7].

In recent years, RBMD has become an increasingly popular trend in unconventional pet food [16] even if raw meat is frequently contaminated with *Salmonella* [22] thereby posing health risks to both animals and owners. A recent Canadian study estimated *Salmonella* prevalence in canine RMBD at approximately 21% with a higher prevalence in raw food diets containing chicken compared with other meat types [23]. Several *Salmonella-*related recalls of raw foods have been reported in recent years, including frozen cat food in the USA [1], in contrast with the decreased *Salmonella* prevalence in dry feeds [24]. Raw food is well-known to pose a substantial risk of infectious disease to the pet, the pet’s environment and the humans in the household, but there are no data on the number of dogs and cats that have become ill after eating contaminated food, or the percentage of pet owners who feed commercial or prepared RMBDs [18]. To date, only one report associated the infection with raw diet and the infection was fatal in cats [9].

Raw food diets for companion animals could represent a potential pet-associated source of *Salmonella* spp*.* in humans. Several reports in the literature have documented *Salmonella* spp. transmission from cats to humans by household and occupational contact [1] but no confirmed cases of human salmonellosis have been associated with raw food diets [23]. In any case, owners who decide to feed animals with RMBD should take strict precautions to avoid direct or indirect transmission, also considering the close relationship that most owners have with their pets.

This report suggests that RMBD should be fed to pets with caution as they could lead to infection, especially in animals with impaired host immune defences. Veterinary practitioners should educate pet owners about the zoonotic risks although the risk to human health remains unquantified.

## Abbreviations

*cpa*: *Clostridium perfringens* toxin A gene
*cpe*: *Clostridium perfringens* enterotoxin gene (*cpe*).
RMBD: Raw meat-based diets
